# Nurses’ Experience in Providing End-of-Life Care in Intensive Care Unit: A Scoping Review

**DOI:** 10.3390/healthcare14030417

**Published:** 2026-02-06

**Authors:** Y. Dodi Setyawan, Indah Ayu Susanti, Cecep Eli Kosasih, Hartiah Haroen

**Affiliations:** 1 Master of Nursing Program, Faculty of Nursing, Universitas Padjadjaran, Sumedang 45363, West Java, Indonesia; dodi25001@mail.unpad.ac.id (Y.D.S.); indah24015@mail.unpad.ac.id (I.A.S.); 2 Department of Critical Care and Emergency Nursing, Faculty of Nursing, Universitas Padjadjaran, Sumedang 45363, West Java, Indonesia; cecep.e.kosasih@unpad.ac.id; 3 Department of Community Health Nursing, Faculty of Nursing, Universitas Padjadjaran, Sumedang 45363, West Java, Indonesia

**Keywords:** ICU nurses, end-of-life care, critical care nursing, nurses’ experiences, scoping review

## Abstract

**Background:** Most ICU patients are in the terminal phase and require complex palliative care support and End-of-Life Care (EoLC). Nurses play a central role in symptom management, emotional support, and shared decision-making. However, evidence on nurses’ experiences in providing EoLC remains fragmented and lacks a comprehensive synthesis. **Objective:** This review aimed to identify, map, and synthesize global evidence on ICU nurses’ experiences in delivering EoLC, including challenges, coping strategies, and implications for critical care nursing practice. **Methods:** A scoping review was conducted following Arksey and O’Malley’s framework and PRISMA-ScR guidelines. Systematic searches were performed in the PubMed, Scopus, and EBSCOhost databases for studies published between 2015 and 2025. Thematic analysis was applied to the qualitative studies to identify patterns and key issues. **Results:** Twelve qualitative studies conducted in diverse countries met the inclusion criteria. Five major themes emerged: (1) emotional and moral challenges; (2) cultural and spiritual influences; (3) communication and interprofessional collaboration; (4) professional development and organizational support; and (5) resource constraints. These findings indicate that ICU nurses’ experiences with EoLC are multidimensional and shaped by the cultural context and institutional policies. **Conclusions:** ICU nurses’ experiences with EoLC reflect complex ethical, emotional, and organizational dimensions. Improving care quality requires structured training, organizational support, and culturally sensitive policies to strengthen critical care nursing practice.

## 1. Introduction

End-of-Life Care (EoLC) in Intensive Care Units (ICUs) has gained substantial global attention over the past two decades. Since 2014, the World Federation of Intensive and Critical Care Medicine has identified EoLC as a strategic priority for addressing existing challenges and promoting the development of regional and national guidelines [1]. Many ICU patients die during their ICU stay, and most hospital deaths occur in these units [2,3]. Considerable variability in EoLC practices, including decisions on limiting life-sustaining treatment, remains evident across countries and healthcare systems, reflecting disparities in economic resources and differences in legal, ethical, and cultural norms [2,4,5].

EoLC in ICUs requires complex symptom management, psychosocial and spiritual support, and shared decision-making among patients, families, and multidisciplinary teams [6,7]. This approach integrates bioethical principles and relies on clear, compassionate communication that reflects patients’ cultural values [6,8]. However, implementation is often hindered by prognostic uncertainty, ethical dilemmas, conflict with families or within teams, and emotional strain among healthcare professionals [9,10]. These challenges can heighten nurses’ moral distress and exacerbate the suffering of patients and their families [8].

Demographic shifts have increased demand for integrated EoLCs in ICUs. Critically ill populations now largely consist of older adults with chronic complex comorbidities [11,12]. While advanced technology has improved survival, it has also intensified dilemmas surrounding aggressive treatments that may prolong the dying process without enhancing quality of life [13,14]. As ICUs increasingly become environments where end-of-life decisions occur, nurses require strong clinical skills, communication capabilities, cultural sensitivity, and interprofessional collaboration to deliver compassionate, patient-centered care [15,16].

Despite their essential role, ICU nurses frequently experience significant psychological, physical, and moral burdens when providing EoLC [17,18]. Moral distress, compassion fatigue, staffing shortages, and insufficient training in end-of-life communication are major barriers to high-quality practice [19,20]. Limited communication failure among professionals and lack of emotional support exacerbate these challenges [21]. Thus, institutional support and continuous education are critical for enhancing nurses’ capacity to deliver effective EoLCs [22,23,24].

Although many qualitative studies have explored ICU nurses’ experiences with EoLC, the existing evidence is fragmented, context-specific, and lacks comprehensive synthesis. Prior reviews often focused on particular regions, narrow aspects of EoLC, or specific clinical populations [25,26]. Consequently, major gaps remain regarding (a) the full spectrum of challenges nurses face, (b) coping or adaptation strategies they use, and (c) enabling factors that support high-quality EoLCs across diverse settings. Additionally, difference in standardized operational guidelines contribute to variability in EoLC practices worldwide. Given these gaps, this scoping review aimed to synthesize scientific evidence on ICU nurses’ experiences in providing EoLC across diverse international contexts. Specifically, it explored the challenges encountered, coping strategies employed, and implications for critical care nursing practice. The findings are expected to guide policy formulation that support patient-centered and culturally sensitive EoLCs.

## 2. Materials and Methods

### 2.1. Study Design

**Figure 1:**
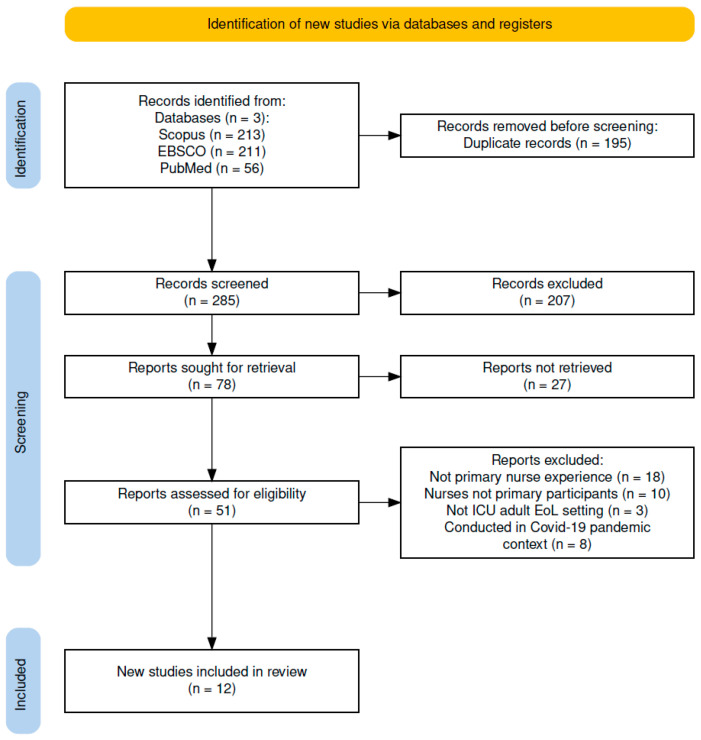
Flow diagram of the literature selection process based on the PRISMA-ScR framework.

This scoping review adopted Arksey and O’Malley’s methodological framework and adhered to the Preferred Reporting Items for Systematic Reviews and Meta-Analyses Extension for Scoping Reviews (PRISMA-ScR) reporting guidelines [27]. The PRISMA 2020 flow diagram is presented in Figure 1, and the completed PRISMA 2020 checklist is provided in the Supplementary Material. This approach was appropriate for exploring and mapping emerging evidence on ICU nurses’ experiences in providing EoLCs. The primary research question guiding this review was How are ICU nurses’ experiences in delivering EoLC reported in international qualitative literature? To ensure transparency, the protocol for this review was retrospectively registered in the Open Science Framework (OSF) (registration DOI: 10.17605/OSF.IO/GWMQS).

### 2.2. Eligibility Criteria

Inclusion criteria were defined using the population–concept–context (PCC) framework. The study population consisted of nurses working in ICUs with at least six months to ≥one year of ICU experience. The concept of this study is the nurses’ experiences related to providing EoLC, including challenges, coping strategies, professional roles, and organizational support. The context of this study was All ICU types across diverse healthcare systems and cultural settings worldwide. Additional inclusion criteria were qualitative or mixed-method studies with identifiable qualitative data, peer-reviewed journal articles, publications in English between 2015 and 2025, and availability of full-text versions. This time frame was selected to capture contemporary evidence reflecting recent developments in ICU practices, evolving ethical frameworks, and current organizational and cultural contexts of end-of-life care.

During full-text screening, we include studies focusing exclusively on end-of-life decision-making without exploring nurses’ experiential perspectives, mixed-methods, studies lacking qualitative components, and studies addressing palliative care in non-ICU settings. Moreover, the exclusion criteria consisted of non-empirical publications (e.g., editorials, commentaries, opinion papers, conference abstracts) and non-peer-reviewed preprints.

### 2.3. Search Strategy

A comprehensive literature search was conducted in PubMed, Scopus, and EBSCOhost (Medline) databases on 6 September 2025. The search strategy combined controlled vocabulary (Medical Subject Headings/MeSH; Cumulative Index to Nursing & Allied Health Literature/CINAHL Headings) and free-text terms by using Boolean operators. The search was limited to studies published between 2015 and 2025, in accordance with the predefined eligibility criteria. Only studies published in English were included because of feasibility constraints. Complete reproducible search strings for each database include: PubMed: ((“Intensive Care Units”[Mesh] OR “Critical Care”[Mesh] OR “intensive care”[tiab] OR ICU[tiab]) AND (“Nurses”[Mesh] OR nurse*[tiab] OR “registered nurse”[tiab]) AND (“Terminal Care”[Mesh] OR “Palliative Care”[Mesh] OR “end-of-life care”[tiab] OR EoLC[tiab]) AND (experience*[tiab] OR perception*[tiab] OR perspective*[tiab])). Scopus: (TITLE-ABS-KEY (“intensive care” OR “critical care” OR ICU) AND TITLE-ABS-KEY (nurse* OR “registered nurse”) AND TITLE-ABS-KEY (“end of life” OR “end-of-life care” OR “palliative care” OR “terminal care”) AND TITLE-ABS-KEY (experience* OR perception* OR perspective*)). EBSCOhost (CINAHL): ((MH “Intensive Care Units” OR “intensive care” OR ICU) AND (MH “Nurses” OR nurse* OR “nursing personnel”) AND (MH “Terminal Care” OR MH “Palliative Care” OR “end-of-life care”) AND (experience* OR perception* OR perspective*)).

### 2.4. Study Screening and Selection Process

Two reviewers (YDS and IAS) independently screened titles, abstracts, and full-text articles using predefined eligibility criteria. The Rayyan.ai was utilized to support the screening process, identify duplicate records, and manage blinded reviewer decisions, whereas Mendeley (version 19.8) was used for reference management. Any discrepancies between the two reviewers were discussed to reach a consensus; when disagreements persisted, a third reviewer (HH) adjudicated the final decision.

### 2.5. Data Extraction and Quality Appraisal

Data extraction was conducted independently by two reviewers (YDS and IAS) using a standardized worksheets. Extracted information included author(s), year of publication, country, study design, participants, ICU setting, and key findings related to ICU nurses’ experiences with EoLC. Any discrepancies in the extracted data were resolved through consensus discussions, and a third reviewer was consulted when agreement was not reached.

### 2.6. Data Analysis and Thematic Synthesis

Thematic synthesis followed the three-stage approach of Thomas and Harden: (1) line-by-line coding, (2) development of descriptive themes, and (3) generation of analytical themes. Two reviewers (YDS and HH) independently conducted line-by-line coding of the findings sections of the included studies. The initial codes were inductively derived and grouped into categories based on conceptual similarity. The categories were iteratively refined into overarching descriptive themes through constant comparisons across studies.

Analytical themes were subsequently generated through interpretive synthesis integrating recurring patterns, contextual nuances, and conceptual relationships beyond the descriptive level. Discrepancies in coding and theme development were resolved through discussion or adjudication by a third reviewer. Rigor was ensured through independent coding, analytic memoing, audit trials, and triangulation across reviewers (YDS, HH).

## 3. Results

### 3.1. Study Selection

The database search yielded 480 records (Scopus, 213; EBSCO, 211; PubMed, 56). After removing 195 duplicate records, 285 unique records were subjected to title and abstract screenings. Of these, 207 were excluded because of irrelevance. A total of 78 full-text articles were retrieved, of which 27 could not be accessed. The remaining 51 full-text articles were assessed for their eligibility. During the 39 full-text screening, studies were excluded for several reasons, including not focusing on ICU nurses’ experiences (*n* = 18), not being the primary study participants (*n* = 10), not being conducted in adult ICU end-of-life care settings (*n* = 3), and being conducted within the COVID-19 pandemic context (*n* = 8). Ultimately, 12 qualitative studies met all inclusion criteria and were included in the review. The complete study selection process is presented in the PRISMA 2020 flow diagram (Figure 1).

### 3.2. Study Characteristics

The 12 included studies were conducted across diverse geographic regions, including Europe (Sweden and Spain), Asia (South Korea, China, and Saudi Arabia), Oceania (New Zealand), the Americas (Canada and Brazil), and Africa (Ghana). All studies utilized qualitative methodologies employing phenomenological, descriptive–interpretive, descriptive–exploratory, or ethnographic designs to investigate ICU nurses’ experiences in providing EoLC. The sample sizes varied widely, ranging from 9 to 100 ICU nurses. Most studies were conducted in adult ICU settings within tertiary or university-affiliated hospitals, although several involved multi-center designs or included specialized units such as general ICUs, neuro, trauma, cardiac, or post-anesthetic care units. Participants predominantly consisted of registered ICU nurses with varied levels of professional experience, and one study specifically focused on male ICU nurses. A summary of the key characteristics of the included studies is shown in Table 1.

### 3.3. Experiences and Challenges of ICU Nurses in Providing EoLC

The thematic synthesis of 12 qualitative studies generated five overarching themes that reflect the emotional, cultural, ethical, and organizational complexities shaping ICU nurses’ experiences in providing EoLC. Table 2 summarizes the major themes and subthemes.

#### 3.3.1. Emotional and Moral Challenges

ICU nurses frequently encounter intense emotional and moral strains while delivering EoLCs. Their close proximity to patient suffering and end-of-life decision-making exposes them to uncertainty, ethical dilemmas, and repeated contact with death, leading to moral distress and value conflict [28,30,35]. Such distress commonly arises when institutional expectations, such as prolonging life-sustaining treatment, conflict with nurses’ clinical judgment and ethical commitment to holistic care [30,32].

Several studies have highlighted that nurses experience profound emotional burdens, including guilt, helplessness, and emotional fatigue, particularly when they perceive treatment as non-beneficial but remain obligated to follow directives shaped by hierarchical decision-making structures [32,37]. Ethical discomfort is heightened during withdrawal of life-sustaining therapy, where nurses feel directly implicated in decisions culminating in patient death, despite limited decision-making authority [37,38].

To manage these pressures, nurses often rely on coping strategies such as emotional distancing or seeking college support, although the effectiveness of such strategies varies widely across clinical environments [28,38]. The evidence collectively indicates that emotional and moral distress is pervasive within the ICU EoLC, reflecting both systemic and interpersonal contributors [37,38].

#### 3.3.2. Cultural and Spiritual Influences

Culture and spirituality strongly shape nurses’ interpretations of death, communication styles, and decision-making during the EoLC. Religious beliefs and cultural norms influence how nurses respond to patient suffering and how they support families in navigating grief and uncertainty [29,33]. In settings where discussing death remains culturally sensitive, nurses may struggle to deliver honest prognostic information, adding emotional complexity to their roles [29,34]. However, providing care to patients from diverse religious backgrounds introduces additional ethical and emotional complexities, requiring nurses to demonstrate high cultural sensitivity, particularly around rituals, family involvement, and expectations for a peaceful death [33,36]. Moreover, cultural diversity within nursing teams creates unique interpersonal dynamics. Nurses who share patient cultural backgrounds often act as informal cultural mediators, which may contribute to cultural fatigue due to repeated cross-cultural role demands [34,36]. Respecting cultural and spiritual values are consistently viewed as essential to safeguard patient dignity [34,39].

#### 3.3.3. Interprofessional Communication and Collaboration

Effective interprofessional communication is foundational for high-quality EoLC, particularly during ethically complex decisions such as withdrawal of treatment [32,37]. However, evidence consistently shows that nurses are frequently marginalized in their decision-making processes. When physicians or families dominate discussions, nurses experience frustration and a sense of powerlessness, as their unique insights grounded in close bedside interactions are not fully acknowledged [5,37]. Additionally, communication barriers and rigid hierarchies intensify moral distress, creating misalignments between medical directives and ethical nursing perspectives [28,32]. These dynamics reduce team cohesion and contribute to emotional conflict during critical care decisions.

Despite these challenges, nurses actively work to promote collaboration by facilitating communication between physicians and families and ensuring clarity and emotional support during patient deterioration [5,28,40]. Their role as communicative bridges helps integrate psychosocial and spiritual needs into medical decision-making. Studies emphasize that open, inclusive communication enhances ethical clarity, strengthens team-based decision-making, and supports patient-centered EoLC [5,32,37].

#### 3.3.4. Professional Development and Organizational Support

Providing EoLCs in ICU settings requires substantial professional competence and psychological resilience. Many nurses, especially those with limited experience, feel inadequately prepared for emotionally intense encounters with patient deaths [34,41]. Targeted training in empathetic communication, reflective practice, and ethical reasoning increases nurses’ confidence, emotional readiness, and ability to provide compassionate care [35,41].

Insufficient organizational support is associated with heightened emotional exhaustion and moral distress. Interventions such as debriefing sessions, peer consultation, and stress management programs contribute significantly to nurses’ psychological resilience and reduce the risk of burnout [30]. These structured supports help nurses process emotional strain and maintain their professional integrity.

Experienced nurses demonstrate a greater capacity for ethical reflection, enabling them to navigate complex moral dilemmas more effectively [5]. Work environments that foster reflective practice and interprofessional dialog enhance both individual well-being and the overall quality of EoLC. Moreover, when organizations provide training, mentorship, and conducive work structures, nurses are better equipped to deliver empathetic patient-centered care. Conversely, a lack of institutional support increases vulnerability to emotional fatigue and diminishes professional meaning [5].

#### 3.3.5. Organizational and Resource-Related Factors

The quality of EoLCs in ICUs is heavily influenced by organizational conditions, including staffing, workload, and clarity of institutional policies. High patient acuity combined with staffing shortages contribute to emotional exhaustion, limited capacity for holistic care, and reduced job motivation among nurses [30]. Resource scarcity, including inadequate equipment and limited physical space, further constrains nurses’ ability to meet patient needs and intensifies feelings of helplessness and moral distress [30,32]. Organizational hierarchies and poor communication structures exacerbate emotional strain, particularly when nurses feel excluded from critical end-of-life decisions [29,32]. Additionally, systemic barriers, such as overcrowded environments, absence of EoLC-specific guidelines, and pressure for maximal medical intervention, complicate the delivery of dignified and comfort-oriented care. Imbalances in interprofessional roles diminish nursing autonomy and elevate work-related stress, undermining collaboration and professional satisfaction [5,29,32].

## 4. Discussion

### 4.1. Main Findings

This scoping review found that the multidimensional challenges experienced by ICU nurses in delivering EoLC. Five overarching themes were identified: (1) emotional and moral challenges; (2) cultural and spiritual influences; (3) interprofessional communication and collaboration; (4) professional development and organizational support; and (5) organizational and resource-related factors.

This review showed emotional and moral challenges emerged consistently as the most prominent themes across the included studies. ICU nurses frequently reported a psychological burden when delivering life-sustaining treatments perceived as non-beneficial or misaligned with patient values. Moral distress, manifested through feelings of guilt, powerlessness, and value conflict, was frequently described in situations where nurses disagreed with the treatment decisions made by physicians or family members [18,42]. Previous evidence further links moral distress with burnout, emotional exhaustion, and decreased retention in critical care environments [18]. Additional literature reinforces these findings, showing that structured interventions such as clinical debriefings, facilitated ethical reflection, and peer support programs can significantly decrease emotional burden and strengthen resilience among ICU nurses [42]. Collectively, these insights emphasize the importance of organizational strategies that acknowledge both the emotional labor and ethical complexities inherent in the EoLC.

The findings of this review underscore the need for cultural humility and integration of spiritual sensitivity into routine EoLC practices. Cultural and spiritual contexts strongly shaped how nurses navigated the EoLC. Religious beliefs, family hierarchies, and culturally grounded expectations about death influence communication, decision-making, and perceptions of a “good death” [28,36]. The Theory of Planned Behavior (TPB) provides a useful interpretive framework, suggesting that attitudes, subjective norms, and perceived behavioral control shape nurses’ intentions and behaviors in EoLC settings [43]. Broader scholarship indicates that cultural norms and family centered decision-making can either support or constrain nurses’ clinical judgment, emotional comfort, and willingness to participate in EoLC discussions.

This review also highlights that effective interprofessional communication is another essential determinant of high-quality EoLC. Most nurses reported being excluded from decision-making processes regarding prognosis, treatment withdrawal, or goals of care despite their central role in bedside monitoring [37,42]. Hierarchical structures, inconsistent communication, and limited interdisciplinary dialog have contributed to frustration and intensified moral distress. Another evidence suggest the benefits of structured communication frameworks, regular interdisciplinary meetings, and family conferences in improving team coordination, clarifying roles, and reducing conflicts [44]. These findings highlight the need to strengthen collaborative structures to meaningfully integrate nursing perspectives into shared decision-making processes.

A consistent finding across studies was nurses’ perceptions of inadequate preparation for the emotional, ethical, and clinical complexities associated with EoLC. Formal training programs, such as the End-of-Life Nursing Education Consortium (ELNEC), have been shown to enhance communication, ethics, and clinical competence [45,46,47]. Complementary approaches, including reflective practice, mentorship, and supportive supervision, can further reduce emotional burden and foster ongoing professional development [48,49]. Therefore, sustained organizational investment in education and support systems is important to promote competence and confidence across all levels of nursing experience.

Institutional factors, including chronic understaffing, high workloads, limited resources, and inconsistent protocols, have a substantial impact on nurses’ EoLC experiences. These challenges often hinder the delivery of compassionate and ethically grounded care [29,30]. Evidence shows that supportive leadership, clear guidelines, collaborative team cultures, and adequate staffing ratios enhance nurses’ perceived behavioral control within the TPB framework, thereby strengthening their confidence and capacity to provide high-quality EoLC [50,51,52,53].

### 4.2. Study Limitations

This study had several limitations. First, a large proportion of the included studies were conducted in Asian and Middle Eastern contexts, which may have introduced cultural bias. Sociocultural norms in these regions, such as hierarchical clinical decision-making, strong family involvement, and culturally specific interpretations of death and dying, may shape nurses’ experiences differently than those in Western or multicultural ICU settings, thus limiting transferability. Second, the evidence base consisted predominantly of qualitative studies, resulting in limited opportunities for methodological triangulation or quantitative comparisons to further validate the identified thematic patterns. Third, although a comprehensive search strategy was applied across the three major databases, restricting the review to English-language publications and excluding inaccessible full texts may have led to language and retrieval bias. Finally, methodological variability across the included studies, such as incomplete reporting of reflexivity, sampling strategies, contextual descriptions, or analytic rigor, may have affected the consistency, depth, and comparability of findings within the synthesis.

### 4.3. Implications for Practice and Research

Reflective and psychologically supportive interventions have been demonstrated to be effective in reducing moral distress and strengthening resilience among ICU nurses. Routine debriefings, ethics rounds, and peer support initiatives facilitate ethical communication, enhance moral courage, and reduce emotional exhaustion [33,54,55]. Moreover, programs such as ELNEC significantly improve nurses’ competencies in communication, symptom management, and ethical decision-making [46,48,56]. Continuous education, simulation-based learning, and technology-enhanced training strengthen self-efficacy and empathy in caring for terminally ill patients [57,58]. Integrating cultural and spiritual competence training ensures holistic and equitable EoLC [59,60]. Cultural reflection-based training and structured interprofessional communication approaches help reduce disparities and enhance the alignment between clinical decisions and patient values [59,61,62]. Simulation-based interprofessional education fosters teamwork and role clarity [63,64]. Incorporating culturally sensitive and spiritually informed care practices improves patients and families [64].

In addition, compassionate and supportive leadership positively influenced the nurses’ well-being and EoLC performance. Perceived organizational support mitigates the impact of workload on burnout [22], while leadership grounded in empathy helps establish a healthy resilience-promoting work culture [65,66]. Moreover, Enhanced interprofessional collaboration and structured family involvement support shared decision-making and improve satisfaction with care [67,68]. Cultural and spiritual competence across healthcare teams further supports sensitive, value-oriented EoLC [39,69,70]. Multidisciplinary spiritual care models show evidence of improved patient quality of life and family experiences [70].

Moreover, these recommendations should follow the future studies to providing evidence-based recommendation. Future studies should evaluate the effectiveness of reflective practices, peer support programs, interprofessional training, and compassionate leadership models on both caregiver and patient outcomes. Longitudinal and cross-cultural research is required to examine the adaptability and impact of moral resilience and cultural competence interventions in diverse healthcare environments. There is also a need to develop validated measurement tools for spiritual competence, inter-professional collaboration, and psychological outcomes among ICU nurses. Mixed-methods study and evaluation designs are recommended to generate context-sensitive evidence-based guidelines for EoLC.

## 5. Conclusions

This scoping review mapped the current evidence on ICU nurses’ experiences in providing EoLCs. The findings indicated that nurses navigate a range of emotional, ethical, cultural, professional, and organizational challenges; however, these experiences vary across settings, cultures, and healthcare systems. This review suggests that emotional burden, moral dilemmas, communication barriers, and cultural considerations are commonly reported, but the extent and expression of these challenges differ among the included studies. This synthesis highlights the potential value of strengthening institutional support, enhancing ethical reflection, and incorporating cultural and spiritual sensitivity into EoLC practices. These insights should be interpreted as indicative rather than definitive patterns given the predominance of qualitative designs and contextual diversity. Future research may benefit from developing and evaluating context-specific interventions, such as structured education, psychosocial support, and culturally responsive interprofessional collaboration, to better understand how these strategies influence nurses’ well-being and quality of care.

## Figures and Tables

**Table 1:** Summary of the characteristics of the studies included in this scoping review.

Author(s), Year	Country	Study Design	Participants	Setting	ICU Type	Data Collection Method	Key Findings
[27]	Saudi Arabia	Qualitative, exploratory–descriptive	10 ICU nurses	Tertiary hospital	Adult ICU	Semi-structured individual interviews	Nurses encountered significant challenges yet remained motivated to deliver high-quality end-of-life care. End-of-life care was provided through holistic, patient-centered approaches addressing physical, emotional, spiritual, and cultural needs. Effective care depended on collaborative teamwork and shared professional ethics within the ICU. Nurses managed complex responsibilities when caring for both dying and critically unstable patients, balancing compassion with clinical demands.
[28]	Brazil	Ethnography (medical anthropology)	10 ICU nurses	Public and private hospitals	Adult ICU	Semi-structured interviews	Decision-making in EOL situations is shaped by the cultural context of the ICU and the social construction of care practices. Nurses’ beliefs, subjective experiences, and professional backgrounds influence how they perceive and engage in EOL decisions-making. Participation in EOL decision is strengthened by professional maturity, communication competence, and the ability to negotiate with both the care team and families. Humanized, patient- and family-centered practices play a central role in guiding ethical and compassionate decision-making.
[29]	Ghana	Qualitative, exploratory	20 ICU nurses	Teaching Hospital	Adult ICU	Semi-structured individual interviews	ICU nurses reported substantial systemic barriers, including inadequate equipment, understaffing, and heavy workloads. Providing EOL care was described as emotionally demanding, associated with significant stress and psychological strain. Nurses frequently encountered family-driven traditional practices that conflicted with medical care and complicated decision-making. Nurses highlighted the need for improved resources, structured communication and education programs, enhanced staff training, and increased workforce capacity.
[30]	South Korea	Qualitative	12 ICU nurses	University Hospital in South Korea	GICU	Focus-group interviews	Nurses perceived end-of-life care as navigating profound human transitions, requiring emotional presence and professional preparedness. Nurses described being deeply involved in patients’ transitions toward death, balancing clinical responsibilities with compassionate support. Professional identity was shaped by the need to be prepared, competent, and resilient when caring for dying patients.
[31]	South Africa	Exploratory–descriptive design	100 ICU nurses	Tertiary public hospitals	Adult ICU	Short survey/ interview guide	Nurses experienced substantial moral distress, primarily driven by collegial incompetence, resource limitations, and complex end-of-life decisions involving the withholding or withdrawal of treatment. Distress was intensified by poor communication, inadequate consultation, and limited opportunities for negotiation within the care team. Participants emphasized the need for stronger support systems, as hierarchical dynamics tied to gender, professional rank, and social status reduced their assertiveness and influence.
[32]	Saudi Arabia	Qualitative, descriptive	8 ICU nurses	Tertiary hospital	Adult ICU	Semi-structured interviews	International ICU charge nurses encountered significant intercultural tensions and misaligned expectations when providing end-of-life care to Muslim patients. Islamic beliefs and strong family involvement profoundly shaped end-of-life decision-making and care priorities. Nurses reported considerable emotional burdens, both personally and within families, during the dying process. Providing care required bridging cultural gaps, honoring spiritual needs, and ensuring peaceful and dignified deaths, whether family members were present or absent.
[33]	Sweden	Qualitative, descriptive	16 ICU nurses	Two university hospitals and two non-university hospitals.	Adult ICU	Individual semi-structured interviews	Nurses perceived patient integrity as central yet difficult to define, often associating it with respect, sensitivity, and patient-centered care. Patient integrity was viewed as vulnerable in the ICU due to patients’ dependence, vulnerability, and the unfamiliar environment; maintaining integrity required protecting patients from harm, respecting individuality, and being attentive to cultural and religious needs.
[34]	Sweden	Qualitative, interpretive–descriptive	20 ICU nurses	Eight ICUs (general + specialized) in Sweden	GICU and specialized ICUs (neuro, trauma, thoracic)	Individual interviews	Nurses encountered ethical tensions when life-sustaining treatments were continued despite minimal chances of survival. Administering pain-relief interventions that could unintentionally hasten death generated moral uncertainty. Ethical conflict emerged when patient wishes to withdraw treatment or family requests to withhold prognostic information diverged from clinical guidelines. Caring for potential organ donors presented dilemmas involving the balance between organ preservation and patient dignity.
[35]	New Zealand	Qualitative	9 ICU nurses	Single-center adult ICU in a large metropolitan hospital providing regional and nationally specialized ICU services	Adult ICU	Semi-structured interviews	Culturally responsive EoLC for Māori relied heavily on acknowledging and involving whānau as integral members of the care team. Nurses experienced both supportive and challenging situations when caring for Māori patients and their families at the end of life. Barriers include limited cultural knowledge, power imbalances, and lack of cultural resources. Facilitators included access to cultural liaisons, supportive EoLC guidelines, and unit-level policies.
[36]	Spain	Qualitative, phenomenological	22 ICU nurses		PARU, GICU, CPCU, and CICU	Semi-structured interviews Unstructured interviews Researcher field notes Participant’ personal letters	Nurses experienced a sense of relief when the limitation of therapeutic effort aligned with patient needs and reduced suffering. They felt compelled to accept physicians’ decisions, even when they were not fully included in the decision-making process.
[37]	Canada	Qualitative, interpretive–descriptive	15 male ICU nurses	Multi-center ICU setting in urban acute care hospitals	Adult Intensive Care Units	Individual interviews	Masculine ideals—such as protectiveness, rationality, and decisiveness—shaped nurses’ end-of-life care practices. Participants frequently expressed emotions openly, challenging traditional masculine norms. Caring for dying patients deepened nurses’ appreciation of personal life and family relationships. Coping strategies commonly included recreation and social support.
[5]	China	Descriptive phenomenological qualitative study	13 ICU nurses	Tertiary hospital	Adult ICU	Semi-structured individual interviews	Nurses described substantial institutional constraints that limited their ability to deliver high-quality end-of-life care. Cultural and cognitive conflicts influenced decision-making processes and complicated interactions with families. Communication breakdowns contributed to a crisis of trust between nurses, physicians, and families.

Note: Intensive Care Units (ICUs); General Intensive Care Unit (GICU); Post-Anesthetic Reanimation Unit (PARU); Cardiac Post-Surgical Care Unit (CPCU); Cardiac Intensive Care Unit (CICU).

**Table 2:** Summary of the main themes and subthemes identified from the thematic analysis of ICU nurses’ experiences in providing EoLc.

Main Themes	Subthemes	Brief Description	Reference
Emotional and moral challenges	Moral distress, emotional burden, value conflicts	Feelings of guilt and helplessness in EoL decision-making	[27,29,31,34,36,37]
Cultural and spiritual influences	Religious beliefs, family values, cultural sensitivity	Influence of spirituality on decision-making and family support	[28,32,33,35]
Interprofessional communication and collaboration	Nursing roles, team dynamics, decision-making conflicts	Team collaboration and open interprofessional communication.	[5,27,31,36]
Professional development and organizational support	Training, mentoring, ethical reflection	Enhancing preparedness and institutional support	[5,29,30]
Organizational and resource-related factors	Limited staffing, EoLC policies, ICU protocols	Impact of policies and resources on EoLC quality	[5,28,29,31]

## Data Availability

No new data were generated or analyzed in this study.

## Supplementary Materials

The following supporting information can be downloaded at: https://www.mdpi.com/article/10.3390/healthcare14030417/s1, File S1: PRISMA 2020 Checklist [71].
